# Editorial: Incretin-based therapies: economic sustainability and health impact in the global management of metabolic syndrome

**DOI:** 10.3389/fphar.2026.1954325

**Published:** 2026-08-17

**Authors:** Alessio Provenzani, Tanja Mueller, Alessandro Mattina

**Affiliations:** 1 IRCCS ISMETT, Palermo, Italy; 2 UPMC Italy, Palermo, Italy; 3 Strathclyde Institute of Pharmacy and Biomedical Sciences, University of Strathclyde, Glasgow, United Kingdom

**Keywords:** cost-effectiveness, GLP-1 receptor agonists, health equity, incretin-based therapies, metabolic syndrome, pharmacoeconomics, tirzepatide, type 2 diabetes

Incretin-based therapies, including glucagon-like peptide-1 receptor agonists (GLP-1 RAs) and dual glucose-dependent insulinotropic polypeptide (GIP)/GLP-1 receptor agonists, have reshaped the management of type 2 diabetes mellitus (T2DM) and obesity through substantial improvements in glycaemic control and body weight, while emerging triple GIP/GLP-1/glucagon receptor agonists are further extending the therapeutic potential of this class (Yao et al., 2024; Bajaj et al., 2026). Large clinical trials have also demonstrated that GLP-1 RAs reduce the risk of major cardiovascular events and adverse kidney outcomes (Badve et al., 2025). Translating these benefits into population-level health gains, however, remains challenging: high acquisition costs and the need for long-term treatment may limit the widespread use of GLP-1 RAs, raising important concerns about affordability and equity, particularly in health systems with limited resources.

The contributions to this Research Topic show that the health impact of incretin-based therapies cannot be understood through efficacy alone, but must be assessed across the diverse clinical and healthcare contexts in which these treatments are used to support personalised and sustainable care.

Three systematic reviews addressed aspects relating to incretin therapies where evidence remains scarce or contested. First, Provenzani et al. focused on solid organ transplant recipients–a population in which post-transplant diabetes and obesity are common but for which tirzepatide data remain extremely limited. In the absence of randomised or interventional trials, the systematic review synthesised four observational studies and reported reductions in HbA1c (−1.4%; 95% CI −1.7 to −0.4) and body mass index (−1.2 kg/m^2^; 95% CI −5.9 to −1.1) with tirzepatide, alongside a low treatment discontinuation rate due to adverse drug reactions (3.1%; 95% CI 0.0–7.1) (Provenzani et al.). Although pooled estimates suggest improvements in metabolic outcomes and acceptable tolerability, the underlying evidence remains limited and vulnerable to residual confounding and selection bias.

Second, Anatriello et al. addressed a question that has attracted increasing regulatory and clinical attention: the ocular safety of GLP-1 RAs (Anatriello et al.). In their meta-analysis of 28 observational studies, GLP-1 RAs were not associated with an increased risk of non-arteritic anterior ischaemic optic neuropathy (NAION) (RR 1.01; 95% CI 0.62–1.64), new-onset diabetic retinopathy (HR 0.96; 95% CI 0.85–1.08), or progression of diabetic retinopathy (HR 0.97; 95% CI 0.83–1.14) compared with other antidiabetic therapies. The analysis also suggested a lower risk of glaucoma, although this finding did not reach statistical significance (HR 0.84; 95% CI 0.71–1.00). However, substantial between-study heterogeneity (I^2^ 65%–91% across outcomes) limits the confidence that can be placed in pooled estimates. This heterogeneity likely reflects differences in study design, outcome definitions, comparator treatments, and follow-up duration, and may also be influenced by residual confounding, channeling bias, and differential ophthalmological surveillance. The NAION estimate in particular appears fragile and dependent on a single study: in a leave-one-out analysis the pooled result reverted to a statistically significant excess risk in the semaglutide group (RR 1.68; 95% CI 1.10–2.57), consistent with the recent European Medicines Agency/Pharmacovigilance Risk Assessment Committee (EMA/PRAC) signal. Continued pharmacovigilance is therefore warranted.

And third, Wu et al. provided the most robust comparative evidence of this Research Topic by conducting a Bayesian network meta-analysis, synthesising 102 randomized controlled trials (98,693 patients) across 15 incretin-based therapies spanning mono-, dual-, and triple-receptor agonists (Wu et al.). Consistent with the trial literature, tirzepatide, orforglipron, and semaglutide ranked highest for glycaemic control (tirzepatide HbA1c mean difference −2.3%), the triple agonist retatrutide was most effective for weight loss (−17 kg), and tirzepatide showed the most favourable cardiovascular profile (MACE odds ratio 0.57; 95% CrI 0.39–0.79). Gastrointestinal adverse events were dose-dependent and hypoglycaemia risk was low, with 45 mg orforglipron offering a favourable efficacy–tolerability balance.

In addition, an economic dimension was directly addressed by Qin et al. who used the validated Building, Relating, Assessing, and Validating Outcomes (BRAVO) microsimulation model to compare a fixed-ratio GLP-1 RA/basal insulin combination (iGlarLixi) with a degludec/aspart co-formulation (IDegAsp) over a 20-year horizon from the perspective of the Chinese healthcare system. Drawing baseline data from the Soli-D trial, the model identified iGlarLixi as a dominant strategy, although the projected health gain was very small (+0.03 QALYs) and cost savings were the principal driver of the result. Total costs were reduced (−US$301), with an 83% probability of cost-effectiveness at a willingness-to-pay threshold of three times *per capita* GDP (≈US$40,344/QALY). The generalisability of these findings beyond the Chinese pricing and healthcare context is, however, limited. Nonetheless, the analysis provides evidence that incretin-containing fixed-ratio combinations may represent economically attractive alternatives to conventional insulin intensification strategies in specific settings.

Taken together, these contributions reinforce the substantial evidence supporting the clinical benefits of incretin-based therapies, while shifting attention to the challenge of translating those benefits across different patient populations and healthcare systems. As effective therapeutic options continue to expand, their broader value will depend on whether they can be used safely in diverse clinical settings and made accessible without placing unsustainable pressure on healthcare resources.

Future research should therefore prioritise pragmatic and real-world studies of access and utilisation, economic evaluations embedded in diverse health systems, and policy analyses addressing reimbursement mechanisms and equitable allocation. Such evidence will be essential to determine whether the substantial metabolic benefits demonstrated in clinical trials can be translated into population-level health gains in a manner that is both sustainable and equitable.

We thank all authors for their contributions and the reviewers for their valuable input, and we hope this collection stimulates the methodological, economic, and policy-oriented research needed to support the responsible global implementation of incretin-based therapies.
